# Perampanel Inhibition of AMPA Receptor Currents in Cultured Hippocampal Neurons

**DOI:** 10.1371/journal.pone.0108021

**Published:** 2014-09-17

**Authors:** Chao-Yin Chen, Lucas Matt, Johannes Wilhelm Hell, Michael A. Rogawski

**Affiliations:** 1 Department of Pharmacology, School of Medicine, University of California Davis, Davis, California, United States of America; 2 Department of Neurology, School of Medicine and Center for Neuroscience, University of California Davis, Sacramento, California, United States of America; CNRS - Université Aix Marseille, France

## Abstract

Perampanel is an aryl substituted 2-pyridone AMPA receptor antagonist that was recently approved as a treatment for epilepsy. The drug potently inhibits AMPA receptor responses but the mode of block has not been characterized. Here the action of perampanel on AMPA receptors was investigated by whole-cell voltage-clamp recording in cultured rat hippocampal neurons. Perampanel caused a slow (τ∼1 s at 3 µM), concentration-dependent inhibition of AMPA receptor currents evoked by AMPA and kainate. The rates of block and unblock of AMPA receptor currents were 1.5×10^5^ M^−1 ^s^−1^ and 0.58 s^−1^, respectively. Perampanel did not affect NMDA receptor currents. The extent of block of non-desensitizing kainate-evoked currents (IC_50_, 0.56 µM) was similar at all kainate concentrations (3–100 µM), demonstrating a noncompetitive blocking action. Parampanel did not alter the trajectory of AMPA evoked currents indicating that it does not influence AMPA receptor desensitization. Perampanel is a selective negative allosteric AMPA receptor antagonist of high-affinity and slow blocking kinetics.

## Introduction

AMPA receptors are members of the ionotropic glutamate receptor family of ligand-gated ion channels [1]. At excitatory synapses throughout the central nervous system, AMPA receptors play a key role as transducers of synaptically released glutamate into fast postsynaptic neuron depolarization. AMPA receptors also are critical to epileptic synchronization and the spread of epileptic seizures, so that pharmacological inhibitors of AMPA receptors have utility in the treatment of epilepsy [2], [3]. The first type of selective AMPA receptor antagonist to be described were competitive antagonists, which bind to the recognition site for glutamate in the ligand binding domain (LBD), stabilizing a closed form of the channel by preventing closure of the clamshell-like LBD [4], [5]. Shortly after the identification of competitive AMPA receptor antagonists, a second type of selective AMPA receptor antagonist was described that acts in a noncompetitive fashion with respect to agonists. These negative allosteric modulators include 2,3-benzodiazepines such as GYKI 52466 [6]–[9] and the related quinazolinone CP-465,022 [10], which bind within peptide segments of AMPA receptor subunits that link the LBD to the transmembrane spanning region [11]. Antagonist occupancy at this site inhibits the transduction of agonist binding into channel gating.

Perampanel [2-(2-oxo-1-phenyl-5-pyridin-2-yl-1,2-dihydropyridin-3-yl)benzonitrile] is a structurally novel AMPA receptor antagonist that is effective in the treatment of partial and secondarily generalized seizures in humans [12], [13]. 2,4-Diphenyl-4*H*-[1], [3], [4]oxadiazin-5-one, the template molecule on which perampanel is based, was discovered by high throughput screening using a rat cortical neuron AMPA-induced cell death assay [14]. Systematic optimization of this template led to the discovery of perampanel, which exhibited high potency as an inhibitor of AMPA-induced Ca^2+^ influx in cultured rat cortical neurons (IC_50_, 0.093 µM) [15]. Studies to date have indicated that perampanel is highly selective. Even a high concentration (30 µM) only minimally inhibits NMDA responses and there is no evidence that perampanel interacts with other ion channel targets. In radioligand binding studies, [^3^H]perampanel binding to rat forebrain membranes was displaced by CP-465,022 and GKYI 52466, indicating that all three agents interact at a common (or allosterically-coupled) site on AMPA receptors. Perampanel has also shown selectivity for AMPA receptor mediated synaptic responses in recordings of field excitatory postsynaptic potentials in the CA1 area in rat hippocampal slices [16]. In these recordings, perampanel inhibited the AMPA receptor component of the field response (IC_50_, 0.23 µM), without affecting the NMDA or kainate receptor components.

While the information available to date is consistent with perampanel acting as a high potency AMPA receptor antagonist, complex effects on the concentration-response curve for AMPA in the Ca^2+^ flux assay have precluded a precise definition of the mode of inhibition [17]. Therefore, the objective in the present study was to characterize the blocking mechanism using whole cell patch clamp techniques. The experimental paradigm also allowed us to define the blocking kinetics. Our results confirm that perampanel inhibits AMPA receptors in a noncompetitive fashion and demonstrate that the onset and recovery of block occurs slowly but is fully reversible.

## Materials and Methods

### Neuronal cultures

All experimental protocols in this work were reviewed and approved by the Institutional Animal Care and Use Committee of the University of California, Davis in compliance with the Animal Welfare Act and in accordance with Public Health Service Policy on the Humane Care and Use of Laboratory Animals.

Primary hippocampal neuronal cultures were prepared as previously described [18]. In brief, timed pregnant rats Sprague-Dawley rats (Charles River Laboratories International, Wilmington, MA, USA) were anesthetized with isoflurane. E18 embryos were obtained and the hippocampi were dissected and treated in Hank’s balanced salt solution (HBSS; Invitrogen, Carlsbad, CA, USA) with trypsin (0.03%, Sigma-Aldrich, St. Louis, MO) for 20 min at 37°C. After inactivation of trypsin with neuronal medium (Neurobasal medium; Invitrogen, Life Technologies, Grand Island, NY, USA) supplemented with NS21, 0.5 mM glutamine, 10 mM HEPES) plus 5% horse serum (HS; Invitrogen), the tissue was washed twice with HBSS, and triturated with a fire-polished Pasteur pipette. After non-dissociated pieces of tissue settled, cells in the supernatant were collected by centrifugation (1100 rpm at 200×g for 3.5 min), re-suspended in neuronal medium plus 5% horse serum, counted, and plated on coverslips (Warner Instruments, Hamden, CT, USA) coated with 0.1% (w/v) poly-L-lysine (Peptides International, Louisville, KY, USA). The cell density was 3×10^4^ cm^−2^ in plating medium. After 4 h, the medium was replaced with serum-free neuronal medium. Cells were grown in a humidified environment of 95% air/5% CO_2_ at 37°C. One third of medium was changed after 5 days in vitro (DIV) and weekly thereafter.

### Voltage-clamp recording

Whole-cell voltage clamp experiments were performed 7–25 days after plating. For AMPA- and kainate-mediated currents, recordings were made in a bath solution containing 135 mM NaCl, 5 mM KCl, 1 mM CaCl_2_, 1 mM MgCl_2_, 10 mM HEPES, 1 µM tetrodotoxin (TTX), 10 µM bicuculline, 1 µM strychnine, and 500 nM MK-801. The pipette solution contained 145 CsCl, 0.1 mM CaCl_2_, 2 mM MgCl_2_, 1 mM EGTA, and 5 mM HEPES. For NMDA-mediated currents, recordings were made in a bath solution containing 135 mM NaCl, 5 mM KCl, 0.2 mM CaCl_2_, 10 mM HEPES, 1 µM TTX, 10 µM bicuculline, 1 µM strychnine, 10 µM NBQX, 3 µM glycine. The pipette solution contained 145 CsCl, 0.1 mM CaCl_2_, 1 mM EGTA, and 5 mM HEPES.

Recordings were made with an Axopatch 200B patch-clamp amplifier (Axon Instruments, Foster City, CA, USA). Whole-cell currents were filtered at 2 kHz and digitized at 10 kHz. All neurons were voltage-clamped at −60 mV. The following drugs dissolved in bath solution were applied using a fast perfusion system (VC^3^-8XP, ALA Scientific Instrument, NY, USA): kainate (3, 10, 100 µM), AMPA (10, 30, 100 µM), NMDA (10, 100 µM), and perampanel (0.01–30 µM). All were from Sigma-Aldrich except perampanel, which was from Eisai Inc. Solution reservoirs contained the drugs separately or the combination of an agonist (kainate, AMPA or NMDA) plus perampanel. Each solution reservoir was connected to a pinch valve of the 8-channel VC^3^-8XP perfusion system, which fed solution to an 8-to-1 mini-manifold (MP-8, Warner Instruments, Hamden, CT) that has a dead-volume of 3.5 µl when switching between solutions. Only one perfusion line was open at a time. The opening speed was 15–20 ms. The command for switching among perfusion lines was generated by the pClamp software and delivered to the control box of the perfusion system via a TTL connection.

### Statistical analysis

Data are expressed as means ± S.E.M. Differences were considered significant at *p*<0.05. The statistical analyses were performed with SigmaStat software (SPSS Inc., Chicago, IL, USA), except that IC_50_ values were compared using SAS software (SAS Institute, Cary NC, USA) as described in the caption to Table 1.

**Table 1:** IC_50_ Values for Perampanel Inhibition of AMPA- and Kainate-Evoked Currents.

Agonist Concentration AMPA/kainate (µM)	AMPA Response		Kainate Response IC_50_ (µM)
	IC_50_, Peak Current, (µM)	IC_50_, Late Current, (µM)	
10/3	0.4	0.4	0.58
30/10	0.8	0.9	0.51
100/100	0.9	1.2	0.58
10 µM vs. 30 µM	*p* = 0.0075	*p*<0.0001	3 µM vs. 10 µM NS
10 µM vs. 100 µM	*p* = 0.0003	*p*<0.0001	3 µM vs. 100 µM NS
30 µM vs. 100 µM	NS	NS	10 µM vs. 100 µM NS

**Figure 1:**
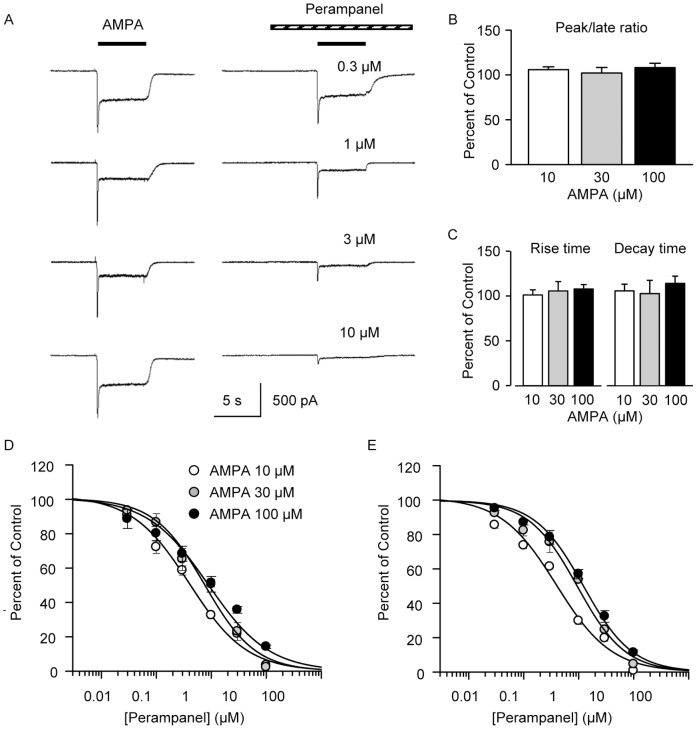
Perampanel inhibition of AMPA-evoked currents in cultured hippocampal neurons. (A) Sample currents evoked by 100 µM AMPA in 4 neurons in the absence (*left panels*) and presence (*right panels*) of perampanel at the concentrations indicated demonstrating a concentration-dependent reduction in current. (B) Perampanel (1 µM) did not alter the mean values of the ratio of the peak to the late amplitude of currents evoked by 10, 30 and 100 µM AMPA in 7, 8 and 7 neurons, respectively, and (C) did not affect the mean rise time constant values or mean decay time constant values of the currents. Peak (D) and late (E) current values evoked by various AMPA concentrations expressed as percent of control prior to perampanel. Curves represent logistic fits to the data for each AMPA concentration. Peak current is the maximum current level; late current is the current during the last 100 ms of the perfusion. Data points in D and E represent mean ± S.E.M. of values from 3 to 9 neurons. Control values for peak/late ratio: 10 µM AMPA: 1.5±0.1; 30 µM AMPA: 1.7±0.1; 100 µM AMPA: 2.7±0.3. Control values for decay time constant (ms): 10 µM AMPA: 141±13; 30 µM AMPA: 93±7; 100 µM AMPA: 50±2. Control values for rise time (ms): 10 µM AMPA: 51±2; 30 µM AMPA: 45±3; 100 µM AMPA: 32±1.

**Figure 2:**
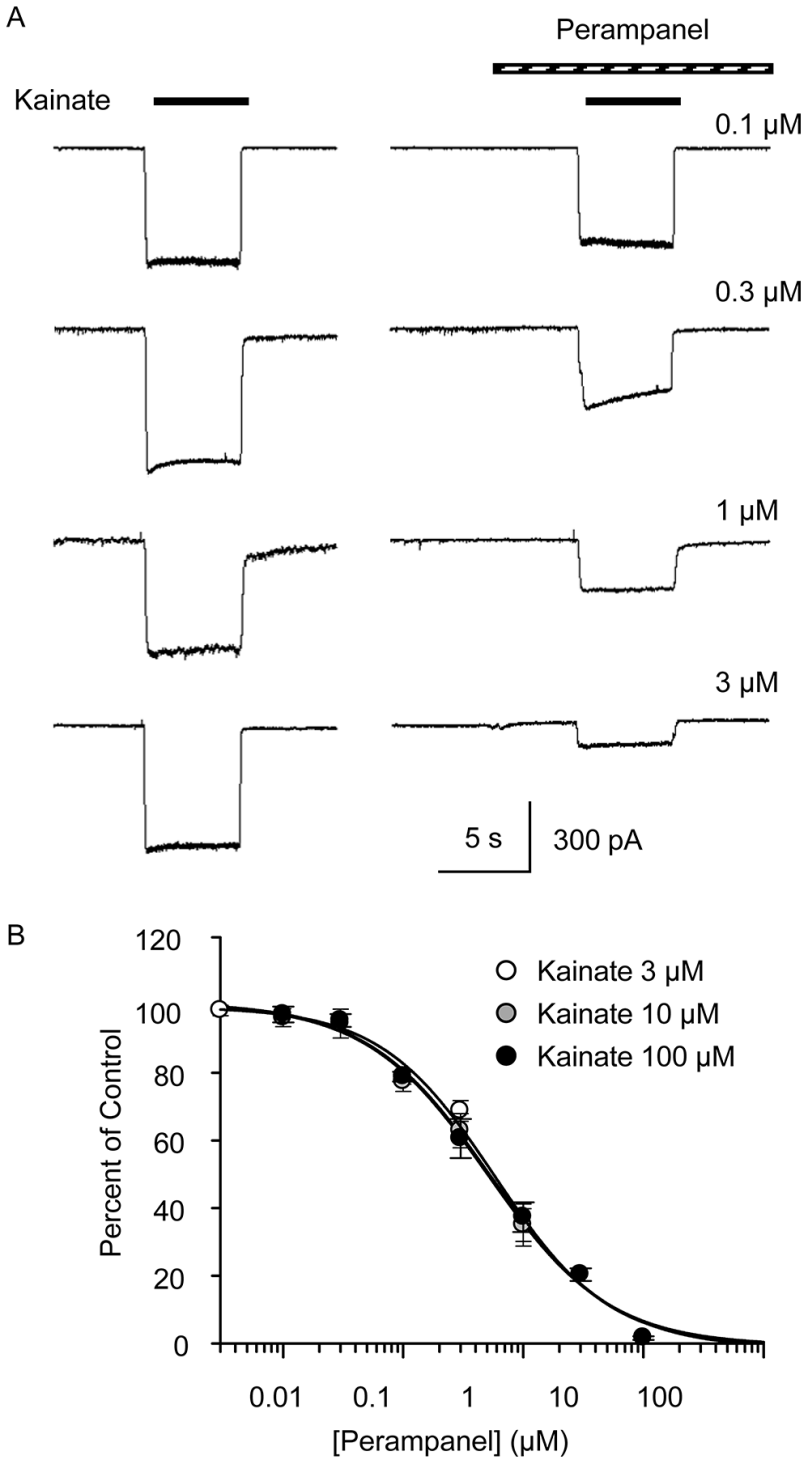
Perampanel inhibition of kainate-evoked currents in cultured hippocampal neurons. (A) Sample currents evoked by 100 µM kainate in 4 neurons in the absence (*left panels*) and presence (*right panels*) of perampanel at the concentrations indicated. The percent inhibition values (derived from the current amplitude in the absence of perampanel and the corresponding current amplitude in the presence of perampanel) are 20%, 41%, 55%, and 80% at concentrations of 0.1, 0.3, 1, and 3 µM, respectively. (B) Current amplitude values evoked by various kainate concentrations expressed as percent of control prior to perampanel. Curves represent logistic fits to the data for each kainate concentration. Data points represent mean ± S.E.M. of values from 3 to 6 neurons.

IC_50_ values are the concentration of perampanel estimated to inhibit the current response by one-half, as determined by logistic fits to the mean percent of control values as presented in Fig. 1D and E, and Fig. 2B. To assess statistical significance of differences between the IC_50_ values, the perampanel concentration-response relationship was linearized. The concentration was log transformed and the current response was transformed as logit Y = ln [(Y+ε)/(Y_max_ – Y+ε)]. Analysis of covariance models were used to estimate IC_50_ values. Differences among the IC_50_ values were tested for significance by comparing the response lines against a common response line, estimated without regard to the concentration. A Tukey-Cramer adjustment was used on those post hoc comparisons. NS, not significant.

## Results

### Perampanel inhibition of AMPA responses

Rapid perfusion of AMPA onto cultured hippocampal neurons at a holding potential of –60 mV elicited an inward current response that decayed rapidly to a steady-state level (Fig. 1A). Preapplication of perampanel for 5 s followed by coapplication of perampanel together with AMPA resulted in a reduction in the amplitude of the peak and late AMPA response, where the late response is taken as the current amplitude at the end of the 5 s AMPA perfusion. The peak and late response amplitude were reduced to a similar extent. This is illustrated in Fig. 1B for recordings in the presence of 1 µM perampanel (which produced near half-maximal inhibition) where the mean ± S.E.M. peak to late ratio was not statistically different from control irrespective of the AMPA concentration. Similarly, as illustrated in Fig. 1C, 1 µM perampanel did not alter the rise time constant or decay time constant at any of the AMPA concentrations. The percent of control peak and late current amplitude values for various perampanel concentrations are plotted in Figs. 1D and E, respectively, for currents evoked by 10, 30 and 100 µM AMPA. Perampanel causes a concentration-dependent inhibition of the peak and late current responses. IC_50_ (half-maximum inhibition) values are 0.4, 0.8, and 0.9 µM for peak AMPA-induced currents for AMPA concentrations of 3, 30 and 100 µM, respectively. The corresponding IC_50_ values for late currents are 0.4, 0.9, and 1.2 µM. As shown in Table 1, there is a statistically significant difference in the IC_50_ values obtained with 10 µM AMPA and those obtained with 30 and 100 µM AMPA. The slight shift in IC_50_ indicates an interaction between perampanel and AMPA binding (see Discussion).

### Perampanel inhibition of kainate responses

Rapid perfusion of 100 µM kainate onto cultured hippocampal neurons at a holding potential of –60 mV elicited a steady inward current response (Fig. 2A). Preapplication of perampanel for 5 s followed by coapplication of perampanel together with kainate resulted in a concentration-dependent reduction in the amplitude of the kainate responses without a consistent alteration in the shape of the responses. The percent of control current amplitude values at various perampanel concentrations are plotted in Fig. 2B for three kainate concentrations (3, 10, and 100 µM). The IC_50_ values are 0.58, 0.51, and 0.58 µM for kainate concentration 3, 10, and 100 µM, respectively. As shown in Table 1, the IC_50_ values are not significantly different.

### Kinetics of perampanel block

**Figure 3:**
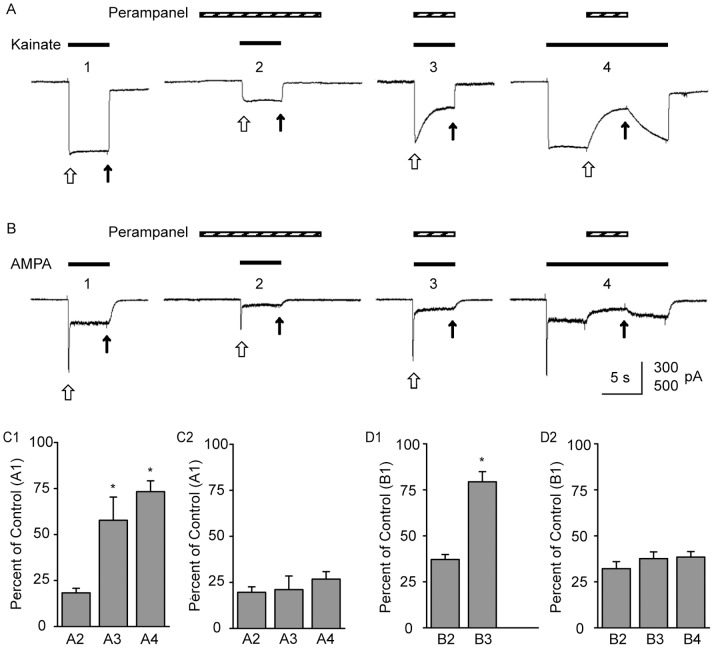
Slow onset and recovery from perampanel block of kainate- and AMPA-evoked currents. (A) Currents evoked by 100 µM kainate in the absence (A1) and with pre-application (A2), co-application (A2) and post-application (A3) of perampanel (3 µM) in the same neuron. (B) Currents evoked by 100 µM AMPA in the absence (B1) and with pre-application (B2), co-application (B2) and post-application (A3) of perampanel (3 µM) in a different neuron from (A). Open arrows indicate peak current levels in the case of AMPA-evoked currents and the current at 100 ms after onset of agonist application in the case of kainate-evoked currents; closed arrows indicate late current levels (end of agonist application). Scale for current is 300 pA in (A) and 500 pA in (B). (C) Mean ± S.E.M. values of peak (C1) and late (C2) current levels as a percent of control (as in A1) with perampanel pre-application (as in A2), co-application (as in A3) and post-application (as in A4) in 3–4 neurons (D) Mean ± S.E.M. values of peak (D1) and late (D2) current levels as a percent of control (as in B1) with perampanel pre-application (as in B2), co-application (as in B3) and post-application (as in B4) in 4 neurons. *p<0.05 vs. A2 or B2.

To assess the rate of perampanel block and unblock we initiated and terminated perampanel fast perfusion in the presence of the agonists kainate and AMPA as illustrated in Fig. 3. Fig. 3A shows sample traces from an experiment with kainate in one neuron. Fast perfusion of kainate 5 s after perampanel (3 µM) perfusion had been initiated resulted in a reduction of the kainate-induced current by ∼73% (Fig. 3A2 versus Fig. 3A1), consistent with the results shown in Fig. 2. Concurrent application of kainate and perampanel (Fig. 3A3) resulted in a decaying current response suggesting the slow development of block over the 5 s combined perfusion. The peak initial current amplitude was modestly reduced from the initial control current amplitude as demonstrated by the summary value (bar A3) in Fig. 3C1. However the magnitude of the late current (bar A3 in Fig. 3C2) amplitude was not different from the magnitude of the late current with perampanel pretreatment (bar A2 in Fig. 3C2). These results demonstrate that perampanel block develops more slowly than channel activation but reaches a plateau that is similar to that achieved with perampanel pretreatment. Similar conclusions can be drawn from the paradigm of Fig. 3A4 where perampanel perfusion is initiated 5 s after the onset of kainate perfusion. The slow development of block (τ = 1.2±0.1 s) is clearly illustrated, as is the slow recovery from block (τ = 3.3±1.5 s).

Similar results were obtained when AMPA was used as the agonist, as illustrated in Fig. 3B. Fast perfusion of AMPA 5 s after the onset of perampanel resulted in reduction in the peak and late responses to a similar extent (∼60%; Fig. 3B2). In contrast, when perampanel and AMPA were applied simultaneously, the peak response was reduced less (17%) than the late response (Fig. 3B3), suggesting that perampanel block is slow to develop. As the extent of late block is similar to the situation with pretreatment, full block is established within 5 s of the recording (Fig. 3D2, compare bar B3 with B2). The slow development of block is confirmed when perampanel perfusion is begun following preexposure to AMPA (Fig. 3B4); this exposure paradigm also demonstrates slow recovery of block of AMPA-evoked current.

**Figure 4:**
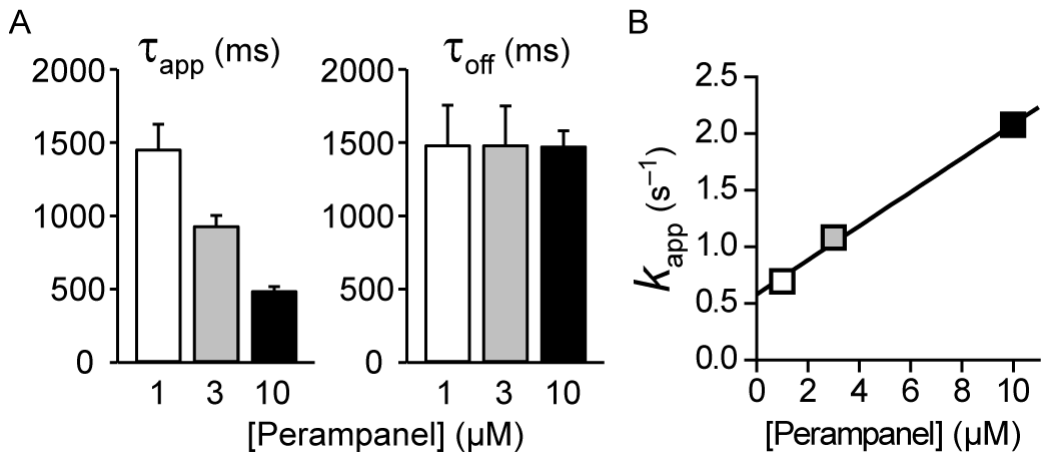
Kinetics of peramapanel block of AMPA-evoked currents. (A) Time constants for block and unblock of 100 µM AMPA-evoked currents determined from the best single exponential fits to current traces at the onset (τ_app_) and termination (τ_off_) of application of 1, 3 and 10 µM perampanel. (B) Reciprocal mean τ_app_ values ( = *k*_app_) plotted against perampanel concentration. The best-fit straight line to the data is shown. The slope and the intercept values are 1.5±0.1×10^5^ M^−1^ s^−1^ and 0.58±0.6 s^−1^, respectively.

The rate constants for binding and unbinding of perampanel were determined from experiments like those shown in Fig. 3B4 with perampanel concentrations of 1, 3 and 10 µM. Apparent rate constants for the approach to equilibrium block (*k*_app_) were calculated as the reciprocal of the time constant values for the onset of block, which are shown in Fig. 4A. As demonstrated by the plot in Fig. 4B, *k*_app_ increases in a linear fashion with increasing perampanel concentration. Assuming a simple one-to-one binding reaction for block, *k*_app_ = *k*_1_[Perampanel]+*k*_–1_, where *k*_1_ and *k*_–1_ are the binding and unbinding rate constants, empirically determined from the best fit straight line to the data as 1.51±0.10×10^5^ M^−1^ s^−1^ and 0.58±0.6 s^−1^, respectively. The mean off rate determined from the τ_off_ values in Fig. 4A is 0.68 s^−1^, which is in good accordance with the value determined assuming a unimolecular binding reaction (intercept of straight line fit in Fig. 4B). The kinetically determined binding affinity (*K*_D_ = *k*_–1_/*k*_1_) is 3.9 µM.

### Perampanel has no effect on NMDA responses

**Figure 5:**
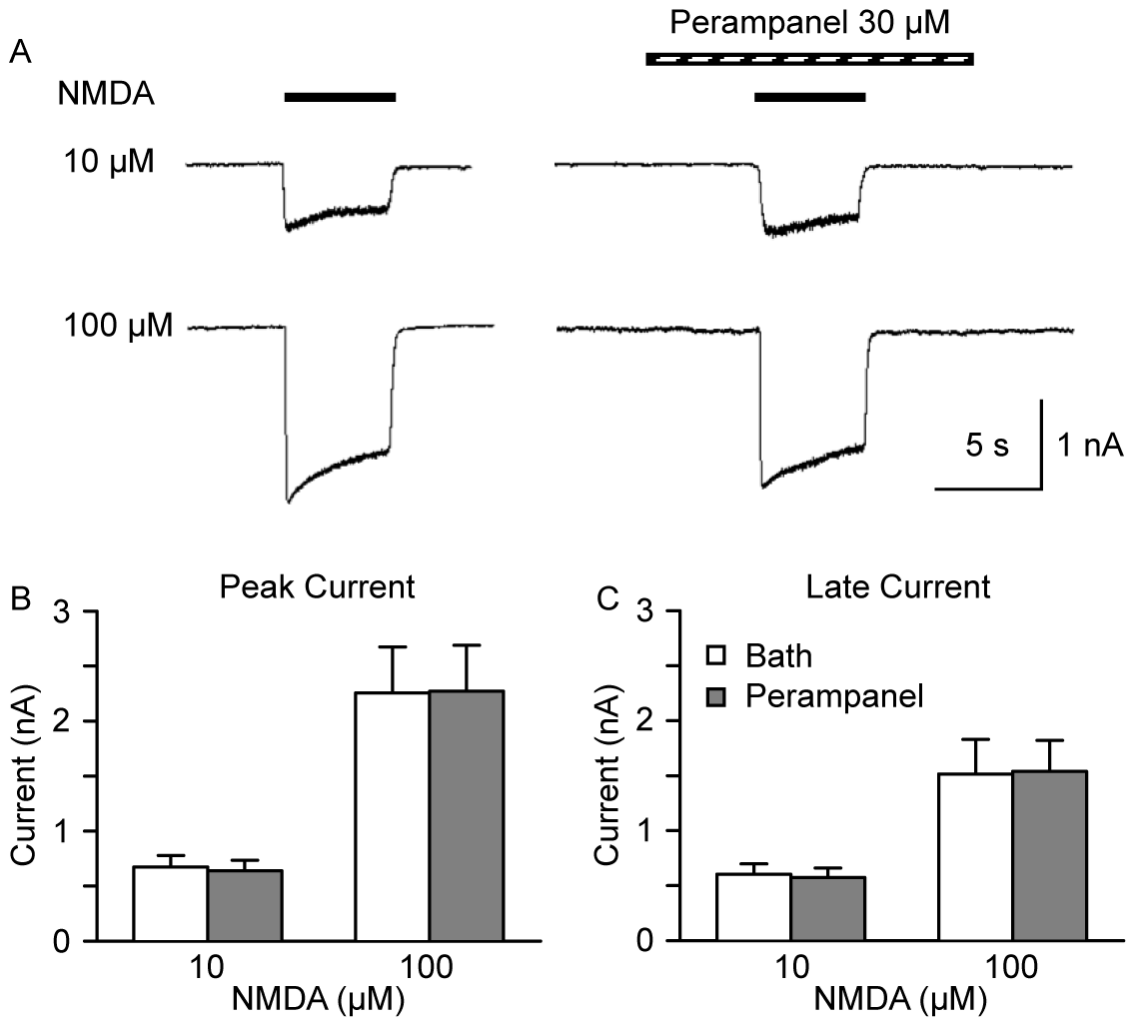
Perampanel does not inhibit NMDA-evoked currents in cultured hippocampal neurons. (A) Sample currents evoked by 10 and 100 µM NMDA in the absence (*left panels*) and presence (*right panels*) of perampanel (30 µM). (B) Mean ± S.E.M. peak amplitude of currents evoked by 10 and 100 µM NMDA during control conditions (bath perfusion) and in the presence of perampanel. (C) Mean ± S.E.M. late amplitude of currents evoked by 10 and 100 µM NMDA during control conditions (bath perfusion) and in the presence of perampanel. Each bar represents data from 6 neurons.

Rapid perfusion of 10 and 100 µM NMDA onto cultured hippocampal neurons at a holding potential of −60 mV elicited inward current responses that decayed modestly during the 5 s perfusion (Fig. 5A). Preapplication of perampanel (30 µM) for 5 s followed by coapplication of perampanel together with NMDA failed to affect the mean amplitudes of the peak (Fig. 5B) or late (Fig. 5C) NMDA-evoked current.

## Discussion

The results of the present study demonstrate that perampanel is a selective noncompetitive AMPA receptor antagonist. In neurons cultured from E18 rat embryos, kainate predominantly activates non-desensitizing or weakly-desensitizing AMPA receptor responses [19]–[22]. In addition, however, in some neurons smaller, rapidly desensitizing currents generated by kainate receptors are present. These currents desensitize completely and the speed of desensitization is so fast (τ ∼ 20 ms) that they do not contribute in a meaningful way to the kainate currents observed in the slower time-scale recordings of the present study. Therefore, for practical purposes, the current responses generated by kainate can be assumed to be mediated by AMPA receptors. Because AMPA receptor currents activated by kainate do not desensitize or desensitize very rapidly to only a limited extent, they are well suited for studies on the mode and kinetics of block since complications caused by desensitization are avoided. In the present study, perampanel blocked kainate-evoked AMPA receptor current to a similar extent irrespective of the kainate concentration, confirming a noncompetitive (allosteric) blocking mechanism. The blocking potency (IC_50_, 0.56 µM) was comparable to that obtained in a previous study in brain slices [16]. The trajectory of kainate-evoked currents was not affected by perampanel. In other words, there was comparable inhibition of the initial current amplitude representing the extent to which closed channels are blocked and the late current amplitude representing the steady-state block of open channels. These findings indicate that perampanel inhibits closed and open channels to a similar extent. This conclusion can be made with confidence inasmuch as perampanel block occurs on a time scale that is much slower (Fig. 3A3) than agonist activation of the channel (Fig. 3A1).

The potency of perampanel block of AMPA receptor responses evoked by a low concentration of AMPA (10 µM) was comparable to the block of kainate-evoked AMPA receptor responses. However, the perampanel dose-inhibition curves were shifted slightly to the right as the concentration of AMPA was increased. Since perampanel block of kainate-evoked AMPA receptor responses did not exhibit this shift, it is unlikely to be due to a competitive blocking interaction. A more plausible explanation is that binding of AMPA transmits a change in conformation to the linker region where perampanel binds, reducing its affinity. Kainate may not induce this effect because it is a partial agonist [20], [23]. Whereas AMPA causes ∼20° of LBD closure which results in strong AMPA receptor desensitization, kainate induces only ∼12° of LBD closure that fails to fully activate the receptor and to desensitize it. AMPA therefore causes greater structural movement than kainate, and the effect of AMPA could be sufficient to influence the interaction of perampanel with its recognition site. In contrast, kainate may not produce a large enough conformational change to influence perampanel binding. An alternative explanation is that the reduced blocking potency relates to reduced affinity of perampanel for desensitized AMPA receptor channels. With AMPA as agonist, due to the rapidity of desensitization, a fraction of the channels even at the onset of perfusion are in the desensitized state [24]. Moreover, the extent of desensitization increases with increasing AMPA concentration [24]. Thus, a reduced affinity of perampanel accompanying desensitization would account for the shift. The apparent more pronounced effect on late than peak current (compare Figs. 1E and 1D) is compatible with this possibility. As is the case for 2,3-benzodiazepine allosteric AMPA receptor inhibitors [6], [24], [25], perampanel did not affect the rate or extent of AMPA receptor desensitization.

A noteworthy observation in the present study is the slow rate at which perampanel block develops and at which unblock from activated receptors occurs. This is demonstrated in the experiments of Fig. 3 where preexposure to perampanel results in fully developed block but without preexposure block develops over the course of ∼3 s. The speed of block is slower than that obtained with 2,3-benzodiazepines. For example, block by GYKI 52466 at a concentration causing ∼50% inhibition of steady AMPA receptor current (30 µM) occurs in several hundred milliseconds [6], [11]. The slower development of block by perampanel is largely due to the slower dissociation rate reflecting higher affinity binding to AMPA receptors. Thus, the association rates for GYKI 52466 [6] and perampanel (this study) are nearly identical at 1.6×10^5^ M^−1^ s^−1^ and 1.5×10^5^ M^−1^ s^−1^, respectively. However, the dissociation rate of GYKI 52466 (3.2 s^−1^) is much faster than that of perampanel (0.58 s^−1^) so that the approach to equilibrium representing the sum of the association and dissociation rates is slower for perampanel.

In sum, the present results support the conclusion that perampanel is a noncompetitive antagonist of AMPA receptor responses that acts in a similar fashion to structurally dissimilar 2,3-benzodiazepine and quinazolinone noncompetitive antagonists. However, perampanel is substantially more potent and exhibits correspondingly slower rates of onset and recovery from block. At steady-state (as is the case during chronic treatment for epilepsy), early and late AMPA receptor responses would be blocked to a similar extent and the block would not be overcome by high synaptic glutamate concentrations as are believed to occur during seizure activity (although there might be a slight effect due to the long distance interaction between binding to the linker and the LBD discussed above). Interictal and ictal epileptiform discharges are much more prolonged than normal synaptic responses; block of late activity is therefore expected to be beneficial in seizure therapy. In contrast, competitive antagonists preferentially block the early component of AMPA receptors current and are less potent inhibitors of late current (see ref. [24]). Unlike the situation for noncompetitive antagonists, the blocking action of competitive antagonists can be overcome by high glutamate concentrations occurring during seizures. Theoretically, therefore, noncompetitive antagonists such as perampanel could have advantages over competitive antagonists in the treatment of epilepsy. In order for competitive antagonists to produce acceptable seizure protection, correspondingly higher doses might be required that would affect normal ongoing synaptic excitation leading to side effects. In fact, the available information indicates that perampanel provides clinically useful seizure protection with an acceptable side-effect profile.
